# Genetic diversity and selection signatures in sheep breeds

**DOI:** 10.1007/s13353-025-00941-z

**Published:** 2025-01-30

**Authors:** Julia Lisboa Rodrigues, Larissa Graciano Braga, Rafael Nakamura Watanabe, Flávio Schramm Schenkel, Donagh Pearse Berry, Marcos Eli Buzanskas, Danísio Prado Munari

**Affiliations:** 1 Departamento de Ciências Exatas, Universidade Estadual Paulista (UNESP), Faculdade de Ciências Agrárias e Veterinárias, Jaboticabal, Brazil; 2 Centre for Genetic Improvement of Livestock, Department of Animal Biosciences, University of Guelph, Guelph, Canada; 3 Animal & Grassland Research and Innovation Center, Moorepark, Fermoy, Co. Cork, Teagasc, Ireland; 4 Departamento de Melhoramento e Nutrição Animal, Universidade Estadual Paulista (UNESP), Faculdade de Medicina Veterinária e Zootecnia, Botucatu, Brazil

**Keywords:** Linkage disequilibrium, Genomics, *Ovis aries*, Selective sweep, Single-nucleotide polymorphism, Site frequency spectrum

## Abstract

Natural and artificial selection in domesticated animals can cause specific changes in genomic regions known as selection signatures. Our study used the integrated haplotype score (iHS) and Tajima’s* D* tests within non-overlapping windows of 100 kb to identify selection signatures, in addition to genetic diversity and linkage disequilibrium estimates in 9498 sheep from breeds in Ireland (Belclare, Charollais, Suffolk, Texel, and Vendeen). The mean observed and expected heterozygosity for all the sheep breeds were 0.353 and 0.355, respectively. Suffolk had the least genetic variation and, along with Texel, had slower linkage disequilibrium decay. iHS and Tajima’s* D* detected selection signatures for all breeds, with some regions overlapping, thus forming longer segments of selection signatures. Common selection signatures were identified across iHS and Tajima’s* D* methods for all breeds, with Belclare and Texel having several common regions under positive selection. Several genes were detected within the selection signature regions, including *ITGA4*, *TLR3*, and *TGFB2* related to the immune system against endoparasites; *DLG1*, *ROBO2*, *MXI1*, *MTMR2*, *CEP57*, and *FAM78B* related to reproductive traits; *WDR70* related to milk traits; *SCHM1* and *MYH15* related to meat traits; and *TAS2R4*, *TAS2R39*, and *TAS2R40* related to adaptive traits. In conclusion, our results demonstrated moderate genetic diversity in the sheep breeds and detected and characterized selection signatures harboring genes associated with reproductive traits, milk production, meat production, and adaptive traits such as endoparasite resistance.

**Supplementary Information:**

The online version contains supplementary material available at 10.1007/s13353-025-00941-z.

## Background

Evolutionary, geographic, and selection processes (natural and artificial) tend to cause specific changes in genomic regions that can control particular traits, such as body conformation and disease resistance (Saravanan et al. 2021). Selection can occur in different forms: positive selection, negative selection, and balancing selection (Nielsen et al. 2005). Positive selection occurs when a particular allele or a mutation confers advantages for a specific trait or the individual’s survival. The frequency of the beneficial allele increases more rapidly than expected compared to a neutral allele thereby contributing to a reduction in genetic diversity and extending the linkage disequilibrium (LD) (Saravanan et al. 2020). Positive selection may also alter the allele frequencies of genetic variants near the location of the beneficial mutation, forming haplotype blocks (Álvarez et al. 2020). Such changes in allele frequency due to positive selection create footprints on the genome known as selection signatures.

Selection signatures can be classified as either a soft sweep when multiple haplotypes flanking the adaptive allele increase to a high frequency in the population or a hard sweep when only a single haplotype with the adaptive mutation increases to a high frequency (Garud 2023). Due to the different types of selection signatures that can be created in the genome, different methods for detecting them have been developed. These methods are used to assess genetic diversity and detect selection signatures in livestock animals, such as cattle (Urbinati et al. 2016), horses (Wolfsberger et al. 2022), fish (Cádiz et al. 2020), goat (Brito et al. 2017a), and sheep (Purfield et al. 2017; Cao et al. 2020; Eydivandi et al. 2021).

High-throughput sequencing and single-nucleotide polymorphism (SNP) genotyping technologies, advanced bioinformatics tools, and robust statistical tests have all created new opportunities to explore and understand genomic selection patterns (Saravanan et al. 2020). The methods to identify selection signatures within populations can be divided into three categories: (1) Site Frequency Spectrum based on the distribution of allele frequencies using statistics like Tajima’s* D* (Tajima 1989) and Fay and Wu’s *H*-statistic (Fay & Wu 2000); (2) LD methods using approaches like Extended Haplotype Homozygosity (EHH) (Sabeti et al. 2002) and Integrated Haplotype Score (iHS) (Sabeti et al. 2007); and (3) methods based on reduced local variability, such as pooled heterozygosity (*H*_P_) (Rubin et al. 2010). Using more than one method can improve the likelihood of detecting selection signatures (Qanbari and Simianer 2014) and also provide confidence in any detected signatures. Nonetheless, because these strategies are based on different statistical assumptions, they can lead to distinct discoveries, which can complement each other.

The identification of selection signatures in commercial sheep breeds is likely due to their high adaptability to different climates, geographic conditions, and diets with poor nutritional quality (Wei et al. 2015; Fariello et al. 2014). Sheep (*Ovis aries*) is a descendant of the Asian mouflon (*Ovis orientalis*), domesticated about 11,000 years ago in Southwest Asia and has reached worldwide distribution (Zeder 2012; Cao et al. 2021). The early selection objectives included behavior toward the presence of humans, not rejecting handling or herding, body size, coat pigmentation, and adaptability (Mirkena et al. 2010). Subsequently, animals started to be selected for specialized production, primarily raised for meat and later for wool, milk, and pelts (Gootwine 2020; Wei et al. 2015) resulting in diverse livestock breeds.

The objective of this study was to explore the genetic diversity and detect and characterize selection signatures in five commercial sheep breeds raised in Ireland. The iHS and Tajima’s* D* methods were used to detect selection signatures leading to and reaching fixation, respectively. In addition, enrichment analysis for the genes identified as being under selection was carried out to provide a clear insight into the genomic mechanisms related to the economic and adaptative traits in sheep.

## Methods

### Genotypic data and quality control

The SNP genotypes used in the present study were from the Irish national sheep breeding program database hosted by Sheep Ireland (http://www.sheep.ie). A total of 50,166 biallelic SNPs from the Illumina OvineSNP50 genotype panel were available for the five breeds of Belclare (*n* = 2444), Charollais (*n* = 1500), Suffolk (*n* = 1242), Texel (*n* = 3399), and Vendeen (*n* = 1114). Phenotypes and pedigree databases were not available for this study. These breeds are included in the Irish breeding program that focuses on meat production traits, such as growth rate and carcass quality (Bohan et al. 2019). All breeds predominate in Ireland, but only the Belclare (a composite breed) originates from the country. Charollais and Vendeen originally come from France, Suffolk from England, and Texel from the Netherlands.

SNP filtering and quality control were performed within each breed using PLINK v1.9 (Purcell et al. 2007) to access the genetic diversity and detect selection signatures. Only SNPs on the autosomes with defined positions according to the sheep (*Ovis aries*) genome assembly 3.1 (OAR 3.1) were considered in the analyses. Samples and SNPs with a call rate below 95% were discarded, as were SNPs with minor allele frequencies below 1% and deviating from the Hardy–Weinberg equilibrium with *p-*value ≤ 10^−6^. The imputation of missing genotypes and haplotype phasing was performed using the software BEAGLE v.3.3.2 (Browning et al. 2018). After quality control, autosomal SNPs on 9498 sheep remained for analyses (Table 1).

**Table 1:** Sample size and number of SNPs before and after quality control

Breed	Purpose	Before quality control		After quality control	
		Sample size	Number of SNPs	Sample size	Number of SNPs
Belclare	Meat and prolific	2444	50,166	2377	30,949
Charollais	Meat	1500	50,166	1477	31,084
Suffolk	Meat	1241	50,166	1231	28,469
Texel	Meat	3399	50,166	3330	30,045
Vendeen	Meat	1114	50,166	1083	29,949

### Genetic diversity metrics

The nucleotide diversity (*π*), inbreeding coefficient (*F*_IS_), observed and expected heterozygosity (*H*_O_ and *H*_E_, respectively), minor allele frequency (MAF), and average pairwise genetic distance (*D*) were calculated for each animal to understand the genetic diversity among and within the five sheep breeds. The *π* was calculated using VCFtools with a 100-kb sliding window (*–window-pi* 100,000) and a step size of 50 kb (*–window-pi-step* 50,000) across the genome. PLINK v1.9 (Purcell et al. 2007) was used to calculate the MAF (option *–maf*), *H*_O_ and *H*_E_ (option *–hardy*), and *F*_IS_ (option *–het*). The *F*_IS_ was calculated as ${F}_{IS}=\frac{{H}_{E}-{H}_{O}}{{H}_{E}}$, based on Wright’s (1950) definition, to measure the excess of homozygotes within each breed. The average proportion of alleles shared between two individuals (*D*_ST_) in a breed was estimated by PLINK v1.9 (Purcell et al. 2007) using the option –*genome*, and the genetic distance between individuals in a sheep breed was calculated as* D* = 1 − *D*_ST_.

### Principal component analysis

Principal component analysis (PCA) was performed using PLINK v1.9 (Purcell et al. 2007) to assess genetic relationships among breeds. Quality control was carried out for all breeds, and SNPs with a call rate below 95% and SNPs with minor allele frequencies below 1% were discarded. After the quality control, 9498 animals and 31,287 SNPs remained for the PCA. Then, the eigenvectors computed for each animal were used to construct dispersion plots along the first two principal components (i.e., PC 1 and PC 2), which explained 36.87% and 16.90% of the total variance for the first and second principal components, respectively.

### Linkage disequilibrium decay

The linkage disequilibrium (*r*^2^) among SNPs was estimated within each sheep breed separately using PLINK v1.9 (Purcell et al. 2007). *r*^2^ quantifies the extent of LD between pairs of loci (Hill 1981). The estimation was performed within non-overlapping windows at 1 Mb, considering all *r*^2^ between markers pairs (*–ld-window-r2* 0). The mean *r*^2^ values were computed within 100-kb intervals, for each breed, spanning the entire 1 Mb distance (Supplementary Table S1).

### Detection of selection signatures

iHS and Tajima’s* D* approaches were used in the present study to increase the ability and power to detect the selection signatures. The integrated haplotype score (iHS) (Eq. 1) is based on the extended haplotype homozygosity (EHH). It compares the integrated EHH profiles between ancestral (0) and derived alleles (1) at a given SNP within a population (Voight et al. 2006). iHS values were calculated with the REHH v.3.2.2 package in R (Gautier & Vitalis 2012; Gautier et al. 2017) within 100-kb non-overlapping windows that had at least two SNPs per window. Extremely negative iHS scores indicate long haplotypes carrying the derived alleles, while extremely positive scores suggest an increase of long haplotypes carrying the ancestral alleles (Voight et al. 2006). The top 1% windows with the highest average |iHS| values were considered regions under positive selection (Voight et al. 2006). The iHS metric was calculated as:

1 $$|iHS|=\frac{ln\left(\frac{{iHH}_{1}}{{iHH}_{0}}\right)- {E}_{P}\left[ln\left(\frac{{iHH}_{1}}{{iHH}_{0}}\right)\right]}{{S}_{P}\left[ln\left(\frac{{iHH}_{1}}{{iHH}_{0}}\right)\right]}$$

where iHH_0_ and iHH_1_ corresponded to the integrated EHH score for ancestral and derived core alleles, respectively; *E*_P_ and *S*_P_ are the expectation and the standard deviation of the |iHS| estimates in frequency bin *p*, respectively.

The Tajima’s* D* statistic (Eq. 2) (Tajima 1989) is based on the Site Frequency Spectrum (SFS) method, which compares the difference between the average number of differences in nucleotides (θ*π*) and the number of segregating sites (θ*s*) estimated from the SNP data (Carlson et al. 2005) and can identify low-to-medium frequency alleles. The Tajimas’* D* values were calculated via VCFtools in sliding windows of 100 kb (*–TajimaD* 100,000). Positive and negative selection reduces heterozygosity and increases the number of rare alleles flanking the selected loci resulting in negative values (Saravanan et al. 2021). Using Tajima’s* D* approach, only negative values were considered, which correspond to recent positive selection signals (Korneliussen et al. 2013; Rajawat et al. 2022). Therefore, the bottommost 1% of empirical Tajima’s* D* values (Korneliussen et al. 2013; Rajawat et al. 2022) that had at least two SNPs per window were considered selection signature regions. Tajima’s* D* metric was calculated as:

2 $${\text{Tajima}}{\prime}\text{s D}= \frac{{\theta }_{\pi }- {\theta }_{\text{S}}}{\sqrt{\text{var }\left({\theta }_{\pi }- {\theta }_{\text{S}}\right)}}$$

### Gene annotation and functional enrichment analysis

The gene annotation and functional enrichment analysis were performed separately, considering one gene list derived from iHS and another from Tajima’s* D* for each sheep breed. Genes and quantitative trait loci (QTL) annotation was performed within the selection signature regions (created after merging the adjacent windows) using the GALLO R package (Fonseca et al. 2020). The ovine reference genome OAR3.1 and the SheepQTL database (https://www.animalgenome.org/cgi-bin/QTLdb/OA/) (Hu et al. 2019) were used in the gene and QTL searches, respectively. The Gene Ontology (GO) and the metabolic pathway retrieved from the Kyoto Encyclopedia of Genes and Genomes (KEGG) database were performed using the gprofiler2 R package (Kolberg et al. 2020; Raudvere et al. 2019). The gene network analysis was undertaken using the STRING software (Szklarczyk et al. 2023). The *p*-values of GO terms and KEGG pathways were adjusted for a False Discovery Rate (FDR) to control for false discoveries (Benjamini & Hochberg 1995), and genes were considered enriched when FDR was less than 0.10.

## Results

### Genetic diversity and population structure

The mean *H*_O_ and *H*_E_ for all breeds were 0.353 and 0.355, respectively **(**Table 2). The Belclare and Vendeen breeds had *H*_O_ values higher than their respective *H*_E_, as evidenced by the negative *F*_IS_ values for both breeds. The values of* D* are a reflection of the homogeneity of animals within each breed, with higher values suggesting greater genetic variation. All breeds had similar* D* values, with the Charollais having the highest. The Suffolk breed had the lowest mean for *π*, *H*o, *H*_E_, and MAF, while the Belclare and Charollais had the highest mean values for *π*, *H*o, *H*_E_, MAF, and *D*.

**Table 2:** Average of the within-breed genetic diversity metrics

Breed (sample size)	Belclare (*n* = 2377)	Charollais (*n* = 1477)	Suffolk (*n* = 1231)	Texel (*n* = 3330)	Vendeen (*n* = 1083)
π*	3.5906	3.6061	3.4430	3.5218	3.4570
*F*_IS_	− 0.0022	0.0014	0.0234	0.0093	− 0.0013
*H*_O_	0.3633	0.3628	0.3386	0.3517	0.3493
*H*_E_	0.3625	0.3633	0.3468	0.3550	0.3489
MAF	0.2767	0.2770	0.2628	0.2698	0.2637
*D*	0.2873	0.2886	0.2794	0.2836	0.2778

*π* nucleotide diversity, *F*_*IS*_ inbreeding coefficient, *H*_*O*_ observed heterozygosity, *H*_*E*_ expected heterozygosity, *MAF* minor allele frequency, *D* average pairwise genetic distance

^*^Values expressed as 10^−3^

### Principal component analysis

**Fig. 1:**
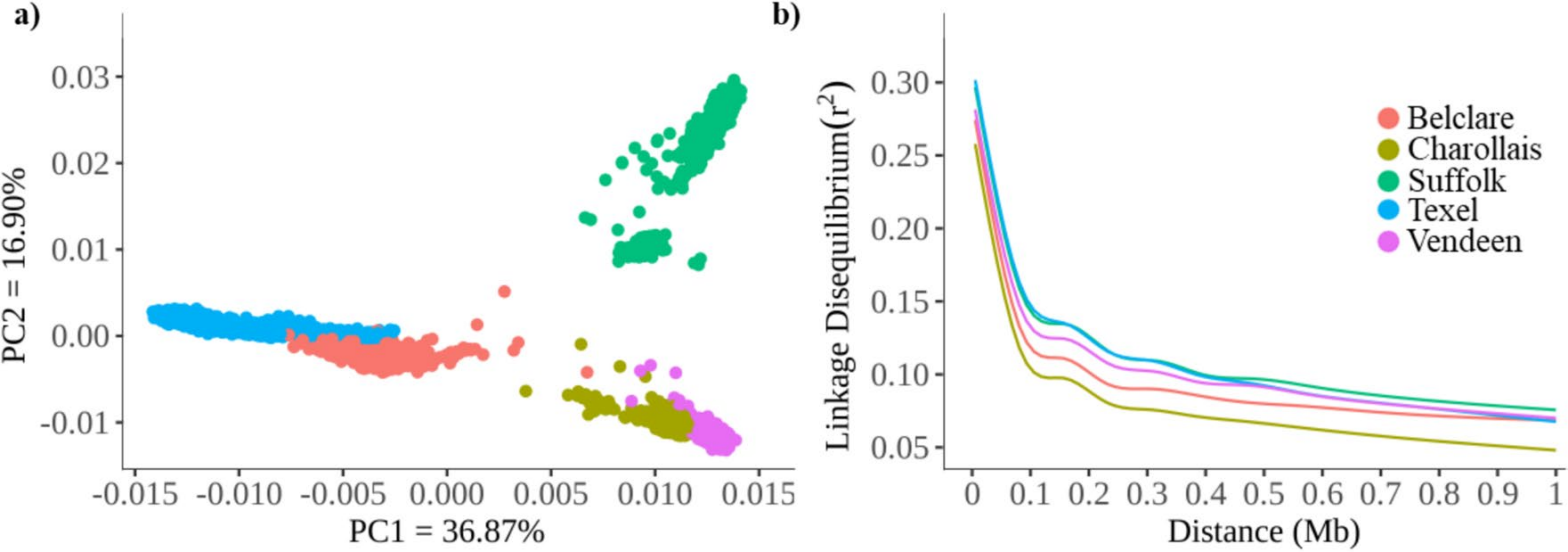
Population genetic analyses of sheep breeds. **a** Principal component analysis of five sheep breeds. **b** Decay of linkage disequilibrium within five sheep breeds

The first four principal components in the PCA (Supplementary Fig. S1) explained 78.24% of the total variance of the population structure among five sheep breeds. The Belclare breed partially overlapped with Texel while the Charollais partially overlapped with Veenden in the PC1 and PC2 (Fig. 1a).

### Linkage disequilibrium decay

The mean *r*^2^ for all sheep breeds within a 1-Mb window was 0.106. The average *r*^2^ at 100 kb for Belclare, Charollais, Suffolk, Texel, and Vendeen breeds was 0.173, 0.157, 0.197, 0.202 and 0.185, respectively (Supplementary Table S1). The overall average among breeds was 0.183. The Suffolk and Texel breeds showed the slowest decay in LD compared to the other breeds (Fig. 1b), as well as they had the greatest number of SNP pairs with *r*^2^ > 0.20 (Supplementary Table S1).

### Selection signatures detection

**Fig. 2:**
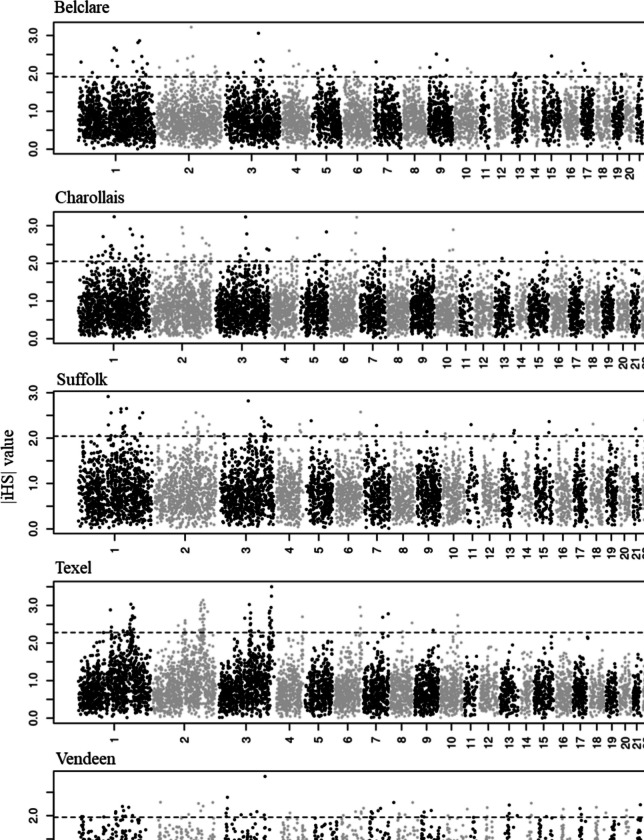
Manhattan plots of iHS values within the 100-kb window in the five sheep breeds. The horizontal line shows the cutoff of the top 1% windows with the highest average |iHS| values that were considered as regions under positive selection

**Fig. 3:**
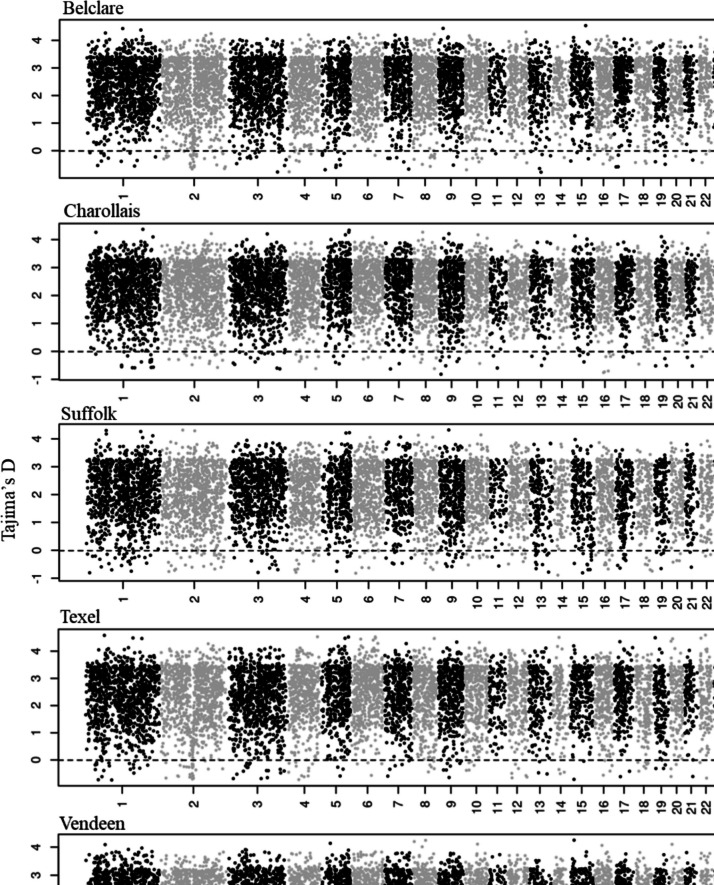
Manhattan plots of Tajima’s* D* values within the 100-kb window in the five sheep breeds. The horizontal line shows the cutoff of the bottommost 1% of empirical Tajima’s* D* values that were considered regions under positive selection

The distribution of |iHS| and Tajima’s* D* values across the autosomes in the five sheep breeds within a 100-kb window is shown in Figs. 2 and 3, respectively. The number of selection signatures regions, genes, and QTL within the positive selection in the five studied commercial sheep breeds is presented in Table 3. The list of genes and QTL harboring the selection signatures is detailed in Supplementary Tables S2 and S3. Selection signature regions overlapped and formed longer segments in all breeds for both methods. Using the iHS method, Belclare, Charollais, Suffolk, and Texel breeds formed selection signatures with lengths of 200 kb, and using Tajimas’* D* method, Belclare and Texel formed signatures with lengths of up to 300 kb (Supplementary Table S4).

**Table 3:** Number of selection signatures (SS^a^) and corresponding genes and QTLs identified within these regions in five commercial sheep breeds using the iHS and Tajima’s* D* methods

Breed	iHS			Tajima’s *D*		
	SS	Genes	QTL	SS	Genes	QTL
Belclare	77	84	162	140	177	218
Charollais	84	88	125	110	145	203
Suffolk	68	85	158	220	255	290
Texel	79	124	96	146	159	240
Vendeen	73	94	185	242	252	259

SS^a^: Top 1% selection signatures regions detected for each sheep breed before merging the adjacent windows

Belclare and Texel breeds had the highest number of common shared regions identified among breeds within each method, with 4 and 21 for iHS and Tajima’s *D*, respectively (Table 4). The common selection signatures regions between the two methods are shown in Table 5. The gene enrichment analyses were performed separately, considering one gene list derived from iHS and another from Tajima’s *D*. The results from the metabolic pathways and Gene Ontology are shown in Supplementary Table S5, and the results from the gene network analyses are shown in Supplementary Figs. S3 and S4.

**Table 4:** Common selection signatures among the five sheep breeds using iHS and Tajima’s methods

Breeds	Method	*N*	Selection signature regions
			(chromosome:position (Mb))
Belclare and Charollais	iHS	2	1:257.3–257.4; 1:243.4–243.5
	Tajima’s* D*	3	2:244.4–244.5; 10:57.6–57.7; 21:26.6–26.7
Belclare, Charollais, and Texel	Tajima’s* D*	3	1:194.6–194.7; 4:41.1–41.2; 7:20.8–20.9
Belclare, Charollais, Texel, and Vendeen	Tajima’s* D*	1	16:36.6–36.7
Belclare, Charollais, and Vendeen	iHS	1	1:148.1–148.2
Belclare and Suffolk	iHS	2	1:256.3–256.4; 18:17.5–17.6
	Tajima’s* D*	7	1:15.3–15.4; 2:213.5–213.6; 2:247.2–247.3; 13:49.2–49.3; 17:8.3–8.4; 20:24.4–24.5; 25:30.0–30.1
Belclare and Texel	iHS	4	1:227.7–227.8; 2:218.4–218.5; 2:129.1–129.2; 3:132.1–132.2
	Tajima’s* D*	21	1:46.3–46.4; 2:21.1–21.2; 2:116.9–117.0; 2:129.4–129.7; 2:131.5–131.7; 2:141.1–141.2; 3:77.4–77.5; 3:90.4–90.5; 3:227.1–227.2; 4:2.4–2.5; 7:12.6–12.7; 8:73.2–73.3; 8:84.7–84.8; 9:75.0–75.1; 12:35.5–35.6; 12:81.7–81.8; 13:43.4–43.5; 16:63.5–63.6; 19:46.1–46.2; 22:29.7–29.8; 25:14.3–14.4
Belclare, Texel, and Vendeen	Tajima’s* D*	2	2:117.6–117.7; 5:74.7–74.8
Belclare and Vendeen	Tajima’s* D*	10	3:25.7–25.8; 3:34.4–34.5; 8:4.0–4.1; 8:68.4–68.5; 8:68.9–69.0; 9:78.6–78.7; 15:1.5–1.6; 15:72.2–72.3; 18:46.3–46.4; 25:30.6–30.7
Charollais and Suffolk	iHS	1	1:193.9–194
	Tajima’s* D*	5	2:122.3–122.4; 2:238.7–238.8; 8:90.8–90.9; 13:57.4–57.5; 16:42.3–42.4
Charollais, Suffolk, and Vendeen	Tajima’s* D*	1	2:29.7–29.8
Charollais and Texel	iHS	3	6:106.1–106.2; 10:69.7–69.8; 10:71.0–71.1
	Tajima’s* D*	3	2:191.0–191.1; 19:39.1–39.2; 24:18.8–18.9
Charollais and Vendeen	iHS	1	1:103.4–103.5
	Tajima’s* D*	12	1:151.5–151.6; 1:185.4–185.5; 1:257.2–257.3; 1:269.3–269.4; 3:158.9–159.0; 4:47.3–47.4; 6:111.6–111.7; 8:28.4–28.5; 9:7.9–8.0; 15:49.2–49.3; 16:52.0–52.1; 16:56.1–56.2; 1:257.2–257.3
Suffolk and Texel	Tajima’s* D*	7	1:177.8–177.9; 1:195.6–195.7; 3:134.8–134.9; 4:26.0–26.1; 6:64.5–64.6; 13:73.4–73.5; 19:8.5–8.6
Suffolk and Vendeen	Tajima’s* D*	15	1:143.1–143.2; 1:212.5–212.6; 1:61.9–62.0; 2:39.1–39.2; 2:78.3–78.4; 8:33.7–33.8; 8:48.3–48.4; 8:48.5–48.6; 9:30.6–30.7; 10:54.0–54.1; 12:17.5–17.6; 13:22.4–22.5; 15:42.3–42.4; 15:61.7–61.8; 17:31.9–32.0
Texel and Vendeen	iHS	1	2:128.7–128.8
	Tajima’s* D*	3	1:219.2–219.3; 2:93.3–93.4; 22:3.5–3.6

*N* number of common selection signatures regions

**Table 5:** Common selection signatures between iHS and Tajima’s methods for each sheep breed

Breed	Chromosome	Position (bp)	Gene
Belclare	3	77,400,000:77,500,000	*SOCS5*
Charollais	1	261,300,000:261,400,000	*CBS, CRYAA, ENSOARG00000011162*
Suffolk	3	23,500,000:23,600,000	*NBAS*
Texel	2	128,400,000:128,500,000	
	2	129,800,000:129,900,000	*LOC101122080*
	3	213,000,000:213,100,000	*ATP6V1E1, ENSOARG0000001347, ENSOARG00000013505*
Vendeen	1	198,000,000:198,100,000	
	1	185,400,000:185,500,000	*SLC49A4*
	1	212,500,000:212,600,000	*SPATA16*
	2	166,000,000:166,100,000	
	2	128,700,000:128,800,000	
	13	22,100,000:22,200,000	*DNAJC1*
	16	14,700,000:14,800,000	*CWC27, SREK1IP1, SHISAL2B, ENSOARG00000025172*

## Discussion

### Genetic diversity

A comprehensive evaluation of genetic diversity across sheep breeds using six parameters: *π*, *H*_*O*_, *H*_E_, *F*_IS_, MAF, and* D* were performed. Among them, MAF is extensively applied in genetic diversity studies to identify rare variants within populations (Bao et al. 2024). In this study, SNPs with an MAF below 0.01 were excluded, as our primary focus was to detect alleles reaching fixation. Consequently, the impact of rare alleles might have been underrepresented. Besides that, filtering SNPs based on an MAF threshold could introduce ascertainment bias, potentially inflating the genetic diversity results, particularly in breeds with lower diversity. However, preliminary analysis evaluated the effect of low-frequency loci (MAF < 0.1) and observed that the removal of these SNP was comparable to the presented results, suggesting that the potential ascertainment bias would likely have minimal impact on our findings. It might be due to the great MAF observed across breeds, with a mean value of 0.27, which aligns with the findings of Heaton et al. (2014), who reported that SNPs with an MAF ≥ 0.3 are highly informative for sheep breeds.

The mean nucleotide diversity for the five sheep breeds in the present study was 3.5 × 10^−3^, comparable to those observed in domestic sheep, whose values ranged from 1.7 × 10^−3^ to 3.1 × 10^−3^ (Lv et al. 2022). Nucleotide diversity is a popular metric to quantify the extent of genetic variation in populations (Wang et al. 2023). Low diversity in sheep is generally associated with a high degree of selection and breeding (Shi et al. 2023), isolation, or small effective population size (Barbosa et al. 2021). In the present study, the breeds were expected to have relatively low diversity, as sheep from Europe have a long history of breed formation and selective breeding (Lv et al. 2022). Belclare is a composite breed and might be expected to have high diversity. However, it experienced intense selection pressure during breed formation, which might explain the low nucleotide diversity values. Regardless of the low values of nucleotide diversity, MAF and* D* in Belclare, Charollais, Suffolk, Texel, and Vendeen variability did exist which suggests that besides the historical formation, the breeds also responded to artificial selection but in different forms due to distinct selection pressures.

The lower genetic diversity in the Suffolk population in the present study, i.e. relatively lower nucleotide diversity, higher inbreeding coefficient, and lower observed heterozygosity, minor allele frequency, and average pairwise genetic distance (Table 2), might be attributable to its small effective population size in Ireland (Purfield et al. 2017). The Suffolk breed imported into Ireland may have passed through genetic bottlenecks resulting from sudden population reduction (Rafter et al. 2022), which was evidenced by Purfield et al. (2017) who reported a greater proportion of the autosomes of Irish Suffolk (which overlapped with those animals used in the present study) covered by short ROH.

The mean *H*_E_ for all the studied breeds was high, which might be due to their breeding history. Similar *H*_E_ estimates were reported for composite and Texel breeds raised in New Zealand with an average of 0.346 (Brito et al. 2017b) as well as in a variety of sheep breeds worldwide, which ranged from 0.22 to 0.38 (Kijas et al. 2012). The sheep breeds with the greatest genetic diversity show lower mean LD and faster LD decay (Kijas et al. 2014). In the present study, the Belclare and Charollais populations had the greatest genetic diversity, the lowest *r*^2^, and a sharp decline in LD (Fig. 1b; Supplementary Table S1), which might indicate a lower level of selection intensity (Brito et al. 2017b). Suffolk and Texel had the least diversity and higher and persistent *r*^2^ over distances of the studied breeds in the present study, indicating they might have experienced the greatest selection pressure over time and/or population bottlenecks.

The high observed heterozygosity in the Belclare breed can be due to their breed formation. Belclare is a composite breed (37.5% Texel, 25.0% Lleyn, 19.5% Finn, 9.0% Galway, 2.5% Cheviot, 1.5% Suffolk, 1.0% Border Leicester, and 4% unknown) produced by crossbreeding to develop animals with a high ovulation rate (Hanrahan, 2024) and good meat quality and production. The cross with Texel was undertaken to select alleles favorable for muscularity, such as the myostatin gene *MSTN* (Purfield et al. 2017). Besides being related to breed formation, the observed higher genetic diversity detected in the Belclare breed might also be due to selection for carcass traits in this breed. (Purfield et al. 2017). In the Charollais breed, a greater nucleotide diversity, observed heterozygosity, minor allele frequency, and average pairwise genetic distance might be due to the higher effective number of founders in this breed (Rafter et al. 2022) and less selection pressure, as evidenced by the lower LD (Fig. 1b).

Demographic history can also explain the diversity observed between and within breeds, and it can be quantified using the PCA. In the present study, PCA revealed genetic similarities between the Belclare and Texel breeds, aligning with the results of O’Brien et al. (2020) who also compared these breeds; this overlap can be attributed to the Texel being used to develop the Belclare breed. The Charollais and Vendeen breeds partially overlapped in the PC1 and PC2, probably because both originated in France; nonetheless, differences between the two breeds in their genetic diversity parameters reflect their different historical selection processes. Suffolk formed two distinct subpopulations in the PCA, which might have been due to the importation of New Zealand Suffolks into Ireland (Featherstone et al. 2021) similar to that documented by O’Brien et al. (2020) also for Irish Suffolks. Nevertheless, maintaining the genetic diversity within breeds is key to breeding programs and conservation strategies; thus, genetic diversity should be monitored over time (Brito et al. 2017b; Rafter et al. 2022).

### Selection signatures and identification of candidate genes

The selection signatures created by natural and artificial selection in sheep can be shared among breeds or be breed-specific, depending on the selection criteria and environment that influenced their breed formation and breeding program. The present study identified common selection signatures between the applied methods, which might provide robust evidence of genomic regions related to the selection process (Shi et al. 2023). Using Tajima’s *D*, we identified a shared selection signature region among Belclare, Charollais, Texel, and Vendeen, which harbored the gene *WDR70* on OAR16. This gene has been previously associated with milk traits in Indigenous sheep breeds (Eydivandi et al. 2021). Although our study breeds are primarily raised for meat production, it might reflect the natural selection pressures related to lamb survival. Belclare, Suffolk, and Vendeen selection signatures regions harbored the gene *SCMH* on OAR1, which was identified as a candidate gene for body weight in Hu sheep (Cao et al. 2020).

Several selection signature regions overlapped, and longer selection signature segments were created in each breed. The extended lengths of haplotypes can indicate regions that have undergone a recent positive selection (Saravanan et al. 2020). Belclare and Texel overlapped the same region (129,400,000–129,700,000, bp) on OAR2 with a 300 kb length, harboring the gene *ZNF385B*. This gene was previously related to live body weight and body conformation traits in Karachai goats (Selionova et al. 2022). The higher number of shared selection signature regions between the Belclare and Texel breeds can be attributed to the historical formation of the Belclare breed, where Texel played an important role, particularly in enhancing meat traits.

Candidate genes associated with meat quality were identified within the selection signatures of Suffolk, including the *MYH15* gene on OAR1 and myosin complex term (GO:0016459) which is involved in muscle fiber and plays an important role in meat tenderness (Yuan et al. 2022). Purfield et al. (2017) identified genes involved in muscle differentiation harboring selection signatures regions on OAR2, such as *ITGAV*, *BIN1*, *MSTN,* and *NUP35* in Irish Belclare and Texel using the Fst and hapFLK methods. Although the present study did not detect these genes, it might be due to the different approaches used to detect selection signatures, so the findings are complementary and help understand the genetic mechanisms related to meat quality traits.

All breeds had selection signatures co-localized in QTL regions for fecal egg count (FEC) trait for the most common nematodes in sheep, *Haemonchus contortus* and *Trichostrongylus colubriformis*. FEC is the most common phenotypic measure used to select sheep resistant to endoparasites (Zvinorova et al. 2016). Several candidate genes associated with resistance to endoparasites were identified within the selection signatures in the present study, including *ITGA4* in Texel, *TLR3* in Suffolk, and *TGFB2* in Vendeen. The genes *ITGA4* and *TLR3* were related to initial rejection and innate immune response infection of *H. contortus* in Florida Native sheep (Estrada-Reyes et al. 2019a). Meanwhile, the *TGFB2* gene was co-localized with selection signatures in resistant sheep breeds and is related to the immune system (Estrada-Reyes et al. 2019b). This might be the result of the artificial selection of resistant sheep, as these breeds are predominantly raised outdoors under grazing systems.

Selection signatures harbored candidate genes related to reproduction ability in ewes in the present study. Reproductive performance plays a crucial role in the profitability of sheep production, as a higher number of lambs born directly enhances farm profitability (Abdoli et al. 2016; Bohan et al. 2019). In Suffolk, the genes *DLG1* and *ROBO2* were identified within selection signature regions and were previously associated with the ovary development process in indigenous sheep breeds (Hernández-Montiel et al. 2020). The gene *MXI1* identified within the selection signature regions in Texel and Belclare plays a positive effect on follicular development in sheep (Wang et al. 2022). Identifying selection signatures regions and genes related to reproduction traits may help to understand the genetic mechanisms involved in this polygenic trait.

Candidate genes related to ram reproduction were also identified within the selection signature regions detected in the present study. Several candidate genes in the Texel breed, such as *MTMR2*, *CEP57*, and *FAM76B*, were previously reported in the Merino sheep breed to be highly expressed in the testicles, acting in sperm motility (Hodge et al. 2023). The genes of the Spermatogenesis-Associated Protein family (*SPATA17* and *SPATA16*) were identified in selection signature regions in Suffolk and Vendeen breeds. These genes play an important role in semen production and semen quality and, consequently, may increase the chance of successful fertilization.

Sheep adaptation to different environments might help them to develop different taste perceptions and food preferences. The type-two taste receptors (*TAS2R*) genes that distinguish bitter-tasting compounds (Henslee et al. 2020), including *TAS2R4* in Texel and *TAS2R39* and *TAS2R40* in Charollais, were identified within the selection signatures in OAR4. These genes have also been previously related to bitter taste in humans, goats, cattle, and dogs (Henslee et al. 2020). The capacity to recognize the bitter taste in grazing animals is associated with a defense strategy against ingesting toxic plants and shrubs (Henslee et al. 2020; Jeruzal-Świątecka et al., 2020). The selective food preferences of sheep might result from evolutionary processes, as their aversion to particular tastes plays a vital role in species adaptation and survival.

A wide range of methods utilizing SNP genotype and sequence data has been developed to detect selection signatures in livestock populations (Qanbari and Simianer 2014). SNP genotype data is widely used in these studies (Geibel et al. 2021). However, the development process of genotyping arrays, designed to detect known polymorphisms, may exclude a proportion of rare alleles, resulting in ascertainment bias (Panigrahi et al. 2023). In our study, quality control was applied to minimize genotyping errors in the data, as it was not possible to determine if the low-frequency genotypes represented new mutations or potential genotyping errors. This process might have excluded some informative SNPs in the studied sheep breeds.

Despite this limitation, the use of two different statistical methods to detect the selection signatures, the detection of overlapping regions, and the use of a large sample size support the reliability of our results. However, analyzing sequence data using the same methodologies used in our study could capture low-frequency alleles and provide more accurate results in genetic diversity metrics, such as heterozygosity and more precise identification of selective signatures due to the higher density and coverage of the genome. Nonetheless, the methodologies employed in our study provide robust results into the genetic diversity and selection signatures for the five sheep breeds from Ireland.

## Conclusion

Overall, moderate genetic diversity was found in the five commercial sheep breeds raised in Ireland. The Suffolk breed was found to have the least genetic diversity and, along with the Texel breed, had the slowest rate of linkage disequilibrium decay among the five studied breeds. Several common selection signature regions were identified across breeds and between the two methodologies used. The genes detected within the selection signature regions included *TLR3* and *TGFB2*, which are related to the immune system against endoparasites; *DLG1*, *ROBO2*, *MXI1*, *MTMR2*, *CEP57*, and *FAM78B* which are associated with reproductive traits; and *TAS2R4*, *TAS2R39*, and *TAS2R40* which are related to adaptive traits. In conclusion, moderate genetic diversity was found in the commercial sheep breeds raised in Ireland. The detected selection signatures harbored genes associated with reproductive traits, milk production, meat production, and adaptive traits such as endoparasite resistance. Therefore, this study contributes to a better understanding of the genetic background of relevant economic and adaptive traits in sheep.

## Supplementary Information

Below is the link to the electronic supplementary material.

Supplementary file1 (DOCX 355 KB)

Supplementary file2 (XLSX 134 KB)

Supplementary file3 (XLSX 113 KB)

Supplementary file4 (XLSX 80 KB)

Supplementary file5 (XLSX 27 KB)

Supplementary file6 (DOCX 1806 KB)

## Data Availability

The genotype data used in this study cannot be made available by the authors as a third party; Sheep Ireland manages them. Reasonable requests for genotype data can be made to Sheep Ireland, Ballincollig, Cork, Co. Cork, Ireland; email query@sheep.ie; Fax + 353 (0)238,820,229; Phone 1,850,601 901; website www.sheep.ie.

## Abbreviations

iHS: Integrated haplotype score
kb: Kilobase
LD: Linkage disequilibrium
MAF: Minor allele frequency
Mb: Megabase
PCA: Principal component analysis
QTL: Quantitative trait loci
SFP: Site frequency spectrum
SNP: Single-nucleotide polymorphism
