# Supplementary material for: Genetic diversity and selection signatures in sheep breeds
Source: J Appl Genet. 2025 Jan 30;66(3):675–87. doi: 10.1007/s13353-025-00941-z (PMC12367903; doi:10.1007/s13353-025-00941-z)
Supplement: Supplementary file 1 — Supplementary file1 (DOCX 355 KB) [file 13353_2025_941_MOESM1_ESM.docx]

**Supplementary Table S1.** Mean linkage disequilibrium (r^2^) for different distances for five sheep breeds.

| **Distance range** | **Belclare** | | | **Charollais** | | | **Suffolk** | | | **Texel** | | | **Vendeen** | | |
| --- | --- | --- | --- | --- | --- | --- | --- | --- | --- | --- | --- | --- | --- | --- | --- |
| **(Mb)** | **Mean** | **NP** | **NP > 0.20** | **Mean** | **NP** | **NP > 0.20** | **Mean** | **NP** | **NP > 0.20** | **Mean** | **NP** | **NP > 0.20** | **Mean** | **NP** | **NP > 0.20** |
| **0-0.1** | 0.173 | 36363 | 10280 | 0.157 | 36524 | 9263 | 0.197 | 31160 | 9998 | 0.202 | 34360 | 11327 | 0.185 | 34034 | 10286 |
| **0.1-0.2** | 0.110 | 41402 | 7391 | 0.096 | 41532 | 6051 | 0.135 | 35328 | 8059 | 0.136 | 39039 | 9071 | 0.124 | 38629 | 7978 |
| **0.2-0.3** | 0.094 | 40791 | 6004 | 0.081 | 41007 | 4703 | 0.115 | 34686 | 6682 | 0.115 | 38509 | 7516 | 0.107 | 38102 | 6669 |
| **0.3-0.4** | 0.087 | 39701 | 5247 | 0.073 | 39819 | 4059 | 0.104 | 33996 | 5788 | 0.104 | 37406 | 6323 | 0.098 | 37342 | 5797 |
| **0.4-0.5** | 0.082 | 34655 | 4139 | 0.069 | 34776 | 3101 | 0.099 | 30719 | 4908 | 0.095 | 33149 | 4986 | 0.093 | 33207 | 4843 |
| **0.5-0.6** | 0.079 | 25795 | 2921 | 0.064 | 26192 | 2047 | 0.093 | 24520 | 3594 | 0.089 | 25604 | 3521 | 0.088 | 25683 | 3384 |
| **0.6-0.7** | 0.075 | 17502 | 1845 | 0.06 | 17651 | 1226 | 0.089 | 17783 | 2459 | 0.082 | 17707 | 2167 | 0.082 | 18064 | 2137 |
| **0.7-0.8** | 0.072 | 11632 | 1120 | 0.057 | 11700 | 722 | 0.084 | 12564 | 1568 | 0.078 | 12076 | 1347 | 0.078 | 12289 | 1345 |
| **0.8-0.9** | 0.072 | 7788 | 768 | 0.051 | 7730 | 425 | 0.079 | 8860 | 1028 | 0.076 | 8206 | 896 | 0.076 | 8130 | 836 |
| **0.9-1.0** | 0.068 | 5253 | 480 | 0.05 | 5276 | 270 | 0.078 | 6093 | 688 | 0.069 | 5633 | 534 | 0.071 | 5495 | 503 |
| **1 Mb** | 0.100 | 260882 | 40195 | 0.086 | 262207 | 31867 | 0.117 | 235709 | 44772 | 0.117 | 251689 | 47688 | 0.110 | 250975 | 43778 |

NP: number of SNP pairs, NP > 0.20: number of SNP pairs with mean linkage disequilibrium higher than 0.20


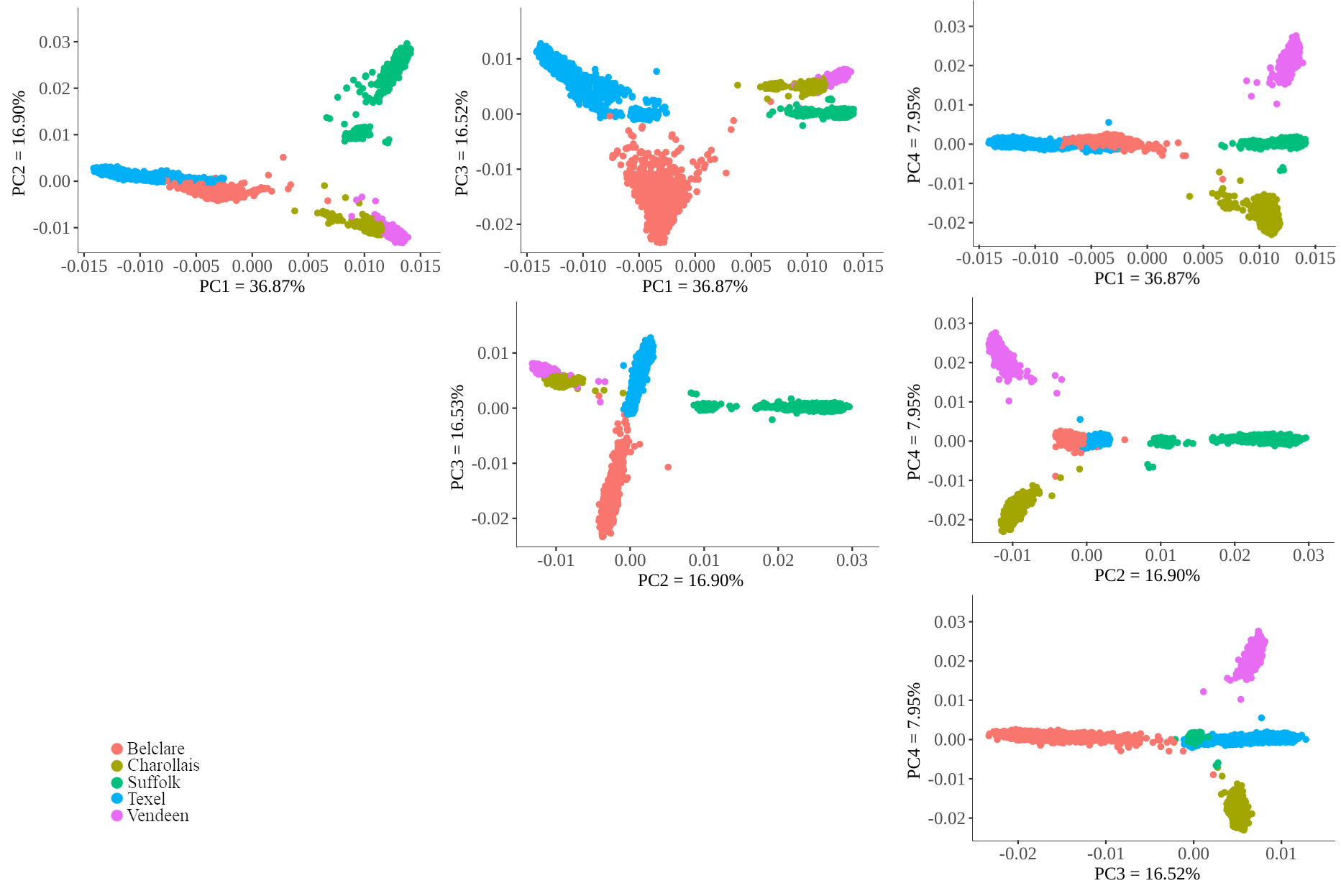


**Supplementary Figure S1.** Principal component analysis of five sheep breeds distributed across the first four principal components.


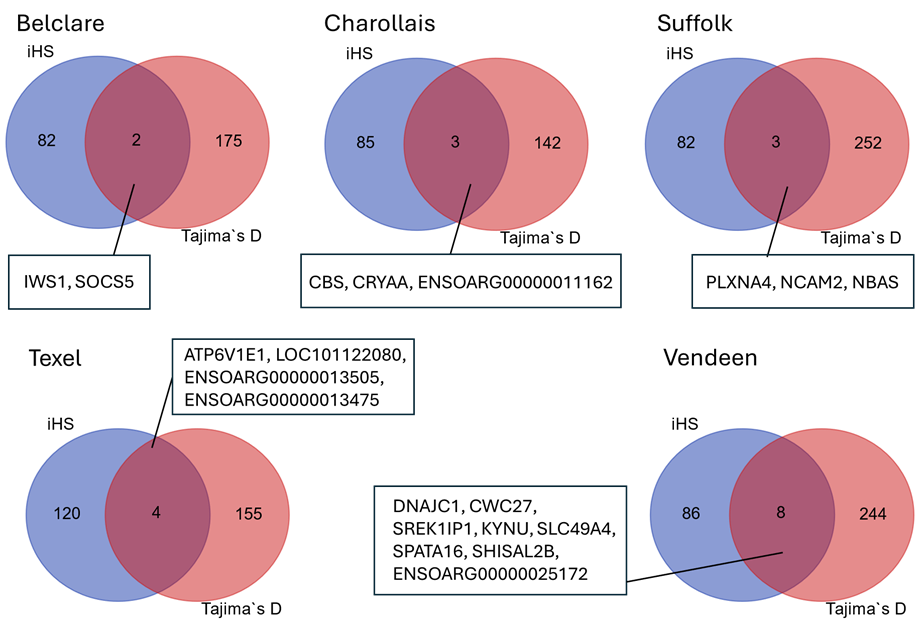


**Supplementary Figure S2**. Common candidate genes detected by iHS and Tajima’s D approach within the selection signatures regions.
