# Supplementary material for: Genetic diversity and selection signatures in sheep breeds
Source: J Appl Genet. 2025 Jan 30;66(3):675–87. doi: 10.1007/s13353-025-00941-z (PMC12367903; doi:10.1007/s13353-025-00941-z)
Supplement: Supplementary file 6 — Supplementary file6 (DOCX 1806 KB) [file 13353_2025_941_MOESM6_ESM.docx]

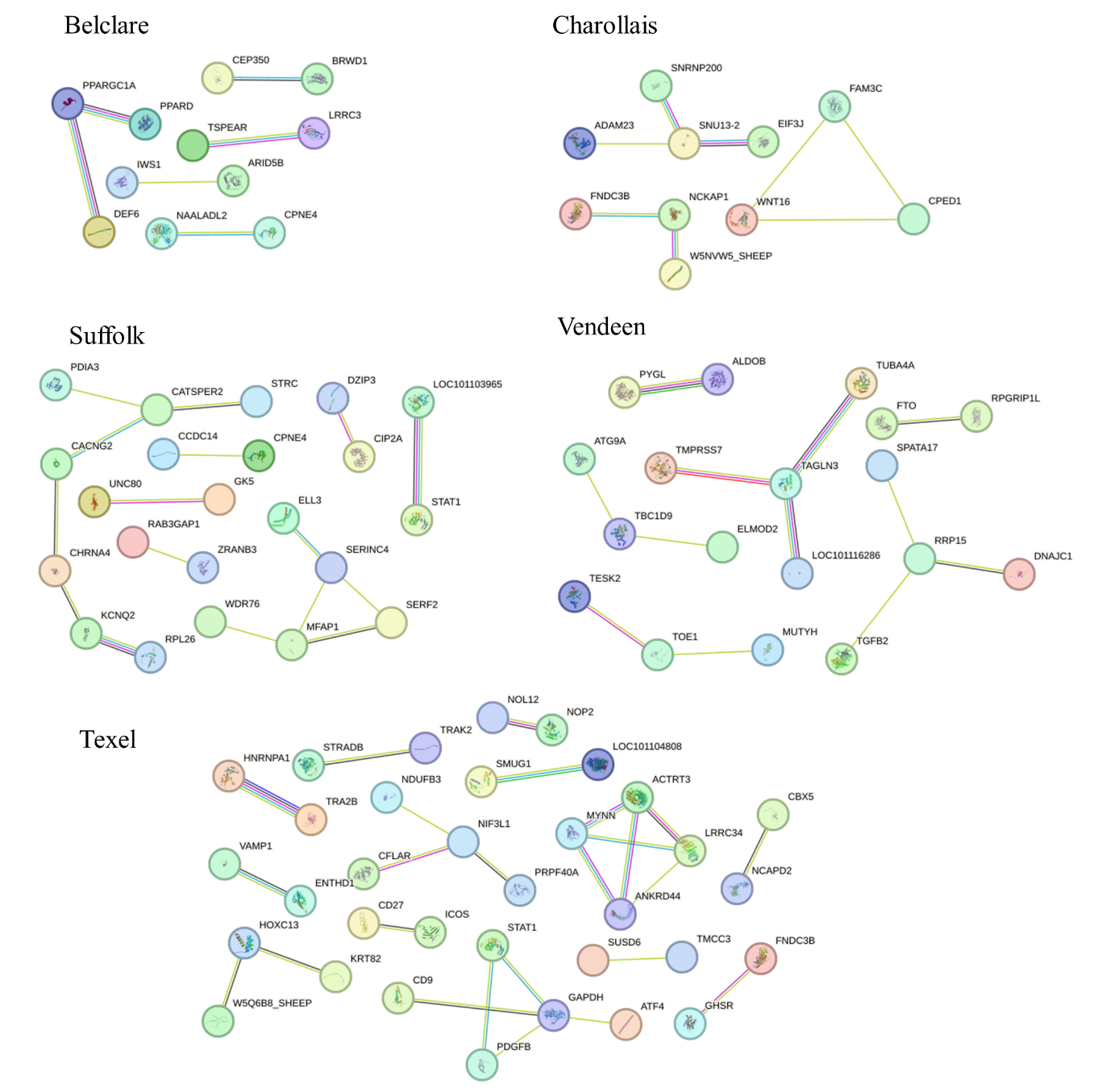


**Supplementary Figure S3.** Network analysis of genes detected by iHS for five sheep populations.


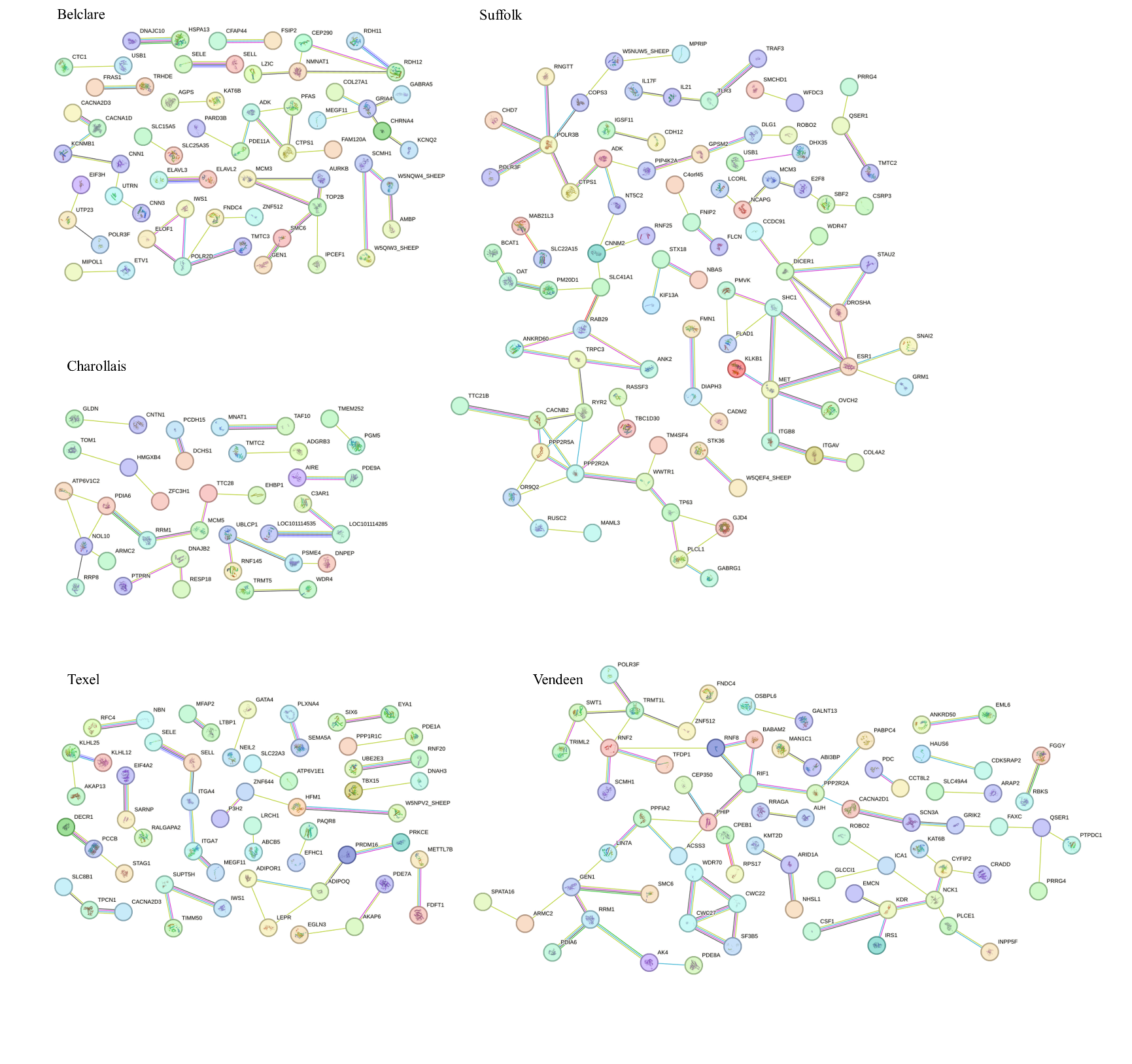


**Supplementary Figure S4.** Network analysis of genes detected by Tajima’s D for five sheep populations.
